# In vivo creatine kinase reaction kinetics at rest and stress in type II diabetic rat heart

**DOI:** 10.14814/phy2.12248

**Published:** 2015-01-27

**Authors:** Adil Bashir, Andrew R. Coggan, Robert J. Gropler

**Affiliations:** Cardiovascular Imaging Laboratory, Mallinckrodt Institute of Radiology, Washington University School of Medicine, St. Louis, Missouri

**Keywords:** ^31^P, creatine kinase flux, diabetic cardiomyopathy, heart, type II diabetes

## Abstract

The effects of type II diabetes on cardiac creatine kinase (CK) enzyme activity and/or flux are unknown. We therefore measured steady‐state phosphocreatine (PCr) and adenosine triphosphate (ATP) content and forward CK reaction kinetic parameters in Zucker Diabetic Fatty (ZDF) rat hearts, a type II diabetes research model. At baseline the PCr to ATP ratio (PCr/ATP) was significantly lower in diabetic heart when compared with matched controls (1.71 ± 0.21 vs. 2.26 ± 0.24, *P* < 0.01). Furthermore, the forward CK reaction rate constant (*k*_*f*_) was higher in diabetic animals (0.52 ± 0.09 s^−1^ vs. 0.35 ± 0.06 s^−1^, *P* < 0.01) and CK flux calculated as a product of PCr concentration ([PCr]) and *k*_*f*_ was similar between two groups (4.32 ± 1.05 *μ*mol/g/s vs. 4.94 ± 1.23 *μ*mol/g/s, *P* = 0.20). Dobutamine administration resulted in similar increases in heart rate (~38%) and *k*_*f*_ (~0.12 s^−1^) in both groups. No significant change in PCr and ATP content was observed with dobutamine. In summary, our data showed reduced PCr/ATP in diabetic myocardium as an indicator of cardiac energy deficit. The forward CK reaction rate constant is elevated at baseline which might reflect a compensatory mechanics to support energy flux through the CK shuttle and maintain constant ATP supply. When hearts were stimulated similar increase in *k*_*f*_ was observed in both groups thus it seems that CK shuttle does not limit ATP supply for the range of workload studied.

## Abstract (precis)

Noninvasive ^31^P MRS was used to measure PCr concentration ([PCr]) and creatine kinase (CK) reaction flux in type II diabetic rat hearts. [PCr] was reduced in diabetic myocardium as compared to controls, indicative of impairment in mitochondrial ATP production. The forward CK reaction rate constant was elevated, possibly reflecting a compensatory mechanism to support increased flux through the CK shuttle required to support cardiac work. CK reaction velocity increased in both diabetic and control hearts to maintain constant ATP content at higher work.

## Introduction

In recent years the number of patients suffering from Type II diabetes mellitus (T2DM) has reached dramatic proportions. Cardiovascular disease is the leading cause of death in diabetic subjects with hypertension and atherosclerosis as the major contributing factors (Haffner et al. 1998; Chen et al. 2011). However, even after controlling for these comorbidities there is an increased incidence of heart failure in diabetic subjects and this is characterized as diabetic cardiomyopathy (DCM) (Bell 2003; Miki et al. 2013). DCM is associated with abnormalities in contractility (diastolic dysfunction which may precede the development of systolic dysfunction) and energy metabolism (Taegtmeyer et al. 2002; Young et al. 2002; Cosson and Kevorkian 2003). In particular, several studies in streptozotocin (STZ)‐treated diabetic rat hearts have reported reduced CK activity and decrease in CK isoenzyme content (Savabi 1988; Popovich et al. 1989; Awaji et al. 1990; Savabi and Kirsch 1991). In contrast Lin et al. (2009) have recently reported reduced levels of phosphorylation of enzyme in diabetic hearts resulting in about 70% increase in the forward CK activity (forward CK reaction is defined as using PCr to produce ATP i.e., PCr‐‐> ATP exchange). Another study has also reported differential modulation of CK activity with diabetes and a 50% increase in CK activity was observed in left ventricles of 8‐week diabetic rat hearts (Somjen et al. 2006). This conflicting data could be due to several reasons; different enzymatic essays used in the studies, reporting of forward versus reverse enzyme activity, severity, duration, and the etiology of diabetes.

Two previous studies employed ^31^P MRS to directly measure the forward CK reaction kinetics in isolated perfused type I diabetic rat hearts (Matsumoto et al. 1995; Spindler et al. 1999). The forward velocity of CK reaction was reduced by about 30% in both the studies. A major limitation of these studies is that the spontaneous heart rate of the perfused diabetic heart was approximately 30% lower than the age‐matched controls and isolated hearts became functionally incompetent when paced at normal rate. Since CK reaction kinetics is closely coupled to the rate of ATP production therefore hearts working at different loads complicates the interpretation of the data from these experiments (Bittl and Ingwall 1985). These studies also reported contradictory results at elevated work presumably due to difficulty in maintaining the viability of isolated diabetic heart.

Spatially localized ^31^P MRS has been used for a long time to noninvasively measure cardiac high‐energy phosphate metabolites in vivo. These studies have been largely limited to the measurement of cardiac PCr/ATP ratios and absolute content (Maslov et al. 2010). Recently rates of ATP synthesis through CK shuttle for in vivo mouse heart have been reported and are significantly reduced in thoracic aortic constriction model of heart failure (Gupta et al. 2011). We have two aims in this study: First is to adapt the ^31^P MRS technique, we had previously developed for human studies, to measure CK flux in rat hearts (Bashir and Gropler 2014). Second aim of the project was to test the hypothesis that in vivo myocardial CK reaction kinetics are reduced in animal model of T2DM. We chose type II diabetes because (1) CK reaction kinetics in type II diabetic hearts has not been studied before, and (2) T2DM accounts for over 90% of diabetes cases in humans. ^31^P saturation transfer measurements were made at rest and during infusion of dobutamine to increase cardiac workload which would yield important information about the CK shuttle response to an adrenergic stress.

## Materials and Methods

### Animal preparation

Animal experiments were approved by the Animal Studies Committee of Washington University in St. Louis and comply with the standards in the Guide and the Animal Welfare Act. Male Zucker Diabetic Fatty (ZDF) rats and their lean littermates were obtained from Charles River Laboratories, Inc. (Wilmington, MA) and maintained on Purina 5008 chow. On this diet, ZDF rats develop diabetes at 12 weeks of age; hence, all studies were done when the rats were 14–15 weeks old. All animals were provided food and water ad libitum at all times except during imaging. The animals were anesthetized using 2% isoflurane, which was maintained throughout the experimental session. Blood glucose levels were assessed with a Bayer Contour blood glucose monitoring system (Bayer HealthCare LLC, Mishawaka, IN). An IV catheter was placed in the tail vein and flushed with heparin/saline solution was used to administer dobutamine. The temperature was maintained by water blanket and constantly monitored during the scan. The heart rate was monitored using an MR‐compatible small animal monitoring and gating System (SA Instruments, Inc.)

### In vivo ^31^P MRS

MRS experiments were performed at 81.5 MHz using an Agilent/Varian 4.7T system (Santa Clara, CA). A 2.4 cm diameter ^31^P surface coil and a geometrically decoupled 4 × 6 cm butterfly proton (^1^H) surface coil were positioned directly beneath the chest of the animal, which was in a prone position. ^1^H MR anatomical images were first acquired to determine positioning of the RF coils. A fiducial attached to the center of the coil was used as a reference to accurately adjust the coil position relative to heart. Adjustment of field homogeneity was performed manually by optimizing the ^1^H signal. The ^31^P signal was localized to the myocardial tissue using the sensitivity profile of the ^13^P surface coil in combination with suppression of signals from superficial (chest) tissues. This was achieved via a modified 1D‐ISIS localization consisting of two scans. In the first scan all the spins within the sensitive region of the RF coil were excited. During the second acquisition the spin population within the superficial tissue was inverted with a B_1_ insensitive adiabatic full passage pulse (AFP) in the presence of linear gradients. After the spins were spatially encoded, a spoiling gradient was applied to dephase any residual transverse component. Addition of the two scans was used to eliminate the signal from selected region. The performance of this approach was tested in a two compartment phantom consisting of stack of 0.5 cm disks containing phenylphosphonic acid (C_6_H_7_O_3_P) and sodium phosphate (Na_2_HPO_4_). Area under the resonances was used to determine the relative phosphate content and signal contamination was determined as the area of the resonance from bottom phantom divided by the total area under the resonances.

In vivo ^31^P MRS data were acquired using a 1 ms 90° adiabatic excitation pulse. Saturation transfer was obtained by low power, narrow band saturation pulse centered on the *γ*‐ATP resonance for 0.2, 0.4, 0.7, 1.6, 2.2, 3, 6, and 9 s. This narrow band pulse directly attenuated the PCr signal by <5%. Data were acquired with an inter‐pulse delay of 9 s and a spectral width of 3000 Hz, with 64 transients acquired for each saturation time. This resulted in a total data acquisition time of about 1.5 h.

Immediately after acquiring the baseline measurements dobutamine was infused, in a subset of animals, using a syringe pump (Harvard Clinical Technology, South Natick, MA) via tail vein catheter at a constant rate of 20 *μ*g/kg/min. ^31^P data acquisition started after a stable heart rate was achieved approximately 5 min after the start of infusion. At the end of experiment animals were allowed to recover in room air and placed in their cages.

### Data analysis

The spectra were processed in the time domain by AMARES (advanced method for accurate, robust, and efficient spectral fitting) algorithm implemented in jMRUI software package (Vanhamme et al. 1997; Naressi et al. 2001). Resonances peaks were fitted to Lorentzian line shapes and soft constraints on the resonance frequencies were used to constrain the fit. For the saturation transfer experiments the PCr line‐width was constrained to the line‐width of control spectra obtained from an unconstrained fit.

Uncalibrated PCr and ATP content in myocardium was measured as the relative area of the respective resonances. Absolute PCr concentration ([PCr]) was calculated making a standard assumption that ATP concentration is 5.5 *μ*mol/g wet weight (8.2 mmol/L cell water) in myocardium (Neubauer 1999; Hitchins et al. 2001; Beer et al. 2002; Kemp et al. 2007; Gupta et al. 2011; El‐Sharkawy et al. 2013). This assumption is commonly used and a range of laboratories and groups that have worked in this area have demonstrated that the myocardial ATP levels remain at normal level until the advanced stages of heart failure (Nascimben et al. 1996; Neubauer 1999). This ATP concentrations are also shown to be remarkably similar in hearts between different animal species and humans despite large differences in size and heart rate. Adenosine Diphosphate concentration ([ADP]) was calculated from CK equilibrium equation by [ADP] = ([TCr]/[PCr] − 1)*([ATP]/(*K*_eq_*H^+^)) where total creatine ([TCr] = [PCr] + [Cr]) is 32 mmol/L, and *K*_eq_ = 1.66 × 10^9^ mol L^−1^ at pH 7 (Ingwall 2002, 2009).

The magnetization of PCr falls exponentially when *γ*‐ATP resonance is saturated and the PCr single intensity was fit to *M(t)* = *M*_*ss*_ + *A.exp(*−*τ.t)* where *τ* = *k*_*f*_ + *R*_1_ (s^−1^) is the apparent relaxation rate constant in presence of exchange and *M*_*ss*_ is the steady‐state magnetization with *γ*‐ATP saturated, *k*_*f*_ (s^−1^) is the forward (PCr to ATP) chemical reaction rate constant of CK and *R*_*1*_ (s^−1^) is the intrinsic relaxation rate constant of PCr in absence of exchange. The CK reaction rate constant was then determined using the following equation
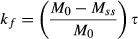


where *M*_o_ is the control PCr intensity in the absence of a saturation pulse (Alger and Shulman 1984; Kuchel 1990).

Data are provided as mean ± SD. Unpaired *t*‐test was performed to test the differences within the groups (rest vs. stress) and between groups (lean vs. obese).

## Results

Characteristics of the control and diabetic rats are given in Table 1. There were no significant differences between the two groups in resting heart rate or bodyweight, although the ZDF rats tended to be a little heavier. The obese rats were hyperglycemic, consistent with their diabetic phenotype. There was a significant increase in heart rate with infusion of dobutamine. The temperature of the animals remained stable during the experiment (data not shown).

**Table 1.** Animal characteristics in Zucker Diabetic Fatty (ZDF) obese and lean counter parts

	Nondiabetic		Diabetic	
	Baseline	Stress	Baseline	Stress
*n*	10	6	10	8
Weight (g)	364 ± 88	387 ± 92	382 ± 42	391 ± 43
Heart Rate (bpm)	314 ± 16*	433 ± 26*	302 ± 30*	415 ± 34*
Blood glucose (mmol/L)	6.69 ± 0.95#	6.57 ± 0.87#	20.20 ± 3.25#	20.94 ± 2.38#

* *P* < 0.05 within group (baseline vs. stress).

# *P* < 0.05 between groups (nondiabetic vs. diabetic).

Values are represented as mean ± SD. *P* < 0.05 was considered significant.

**Figure 1.**
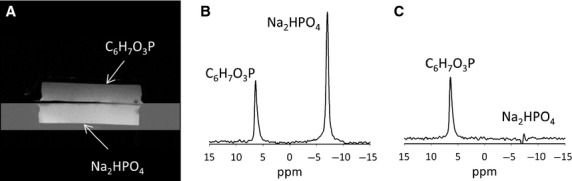
(A) ^1^H reference MR image of the two compartment phantom. Each compartment is about 0.5 cm deep. The RF coil was placed directly below the phantom. (B) ^31^P spectrum acquired in the absence of the suppression band showing distinct resonances from the two phosphate solutions. The Na_2_HPO_4_ resonance is larger as it is closer to the RF coil. (C) Example of spectrum acquired when the suppression band is placed on the bottom phantom containing Na_2_HPO_4_. The resonance signal from Na_2_HPO_4_ is almost completely eliminated.

**Figure 2.**
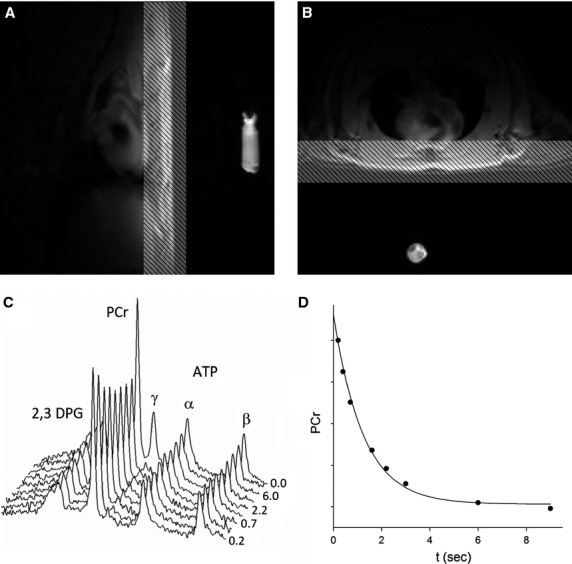
(A) Sagittal and (B) axial reference images of the rat heart. A small fiducial indicating the coil center is visible in the images. Images also show the placement of saturation band. (C) Stacked plot of spectra is displayed. The duration (s) of saturation of *γ*‐ATP is shown on the right. For clarity duration is displayed for every alternate spectrum. *γ*‐ATP is visible in the spectrum when no saturation is applied (*t* = 0 s). 2,3‐diphosphoglycerate (2,3 DPG) resonance arises from the blood in myocardium and other abbreviations are defined in the main text. (D) Example demonstrating the fitting of the saturation transfer equation to the PCr resonance area.

Figure 1 shows the MR image of the two compartment phantom and spectra acquired in the absence (Fig. 1B) or presence (Fig. 1C) of the saturation band. Spectra show that the resonance from sodium phosphate was eliminated when the saturation region was placed on the bottom compartment. The signal contamination, expressed as a percentage of the total signal, was <3% for a typical experiment. Figure 2A and B show high‐resolution sagittal and axial images of the heart obtained using the ^1^H surface coil. ^31^P spectra from heart under different saturating conditions is shown in Figs 2C and PCr resonance amplitude diminishes as the duration of *γ*‐ATP saturation is increased. Figure 2D shows a graph of PCr resonance intensity versus saturation time and the resulting exponential fit.

**Figure 3.**
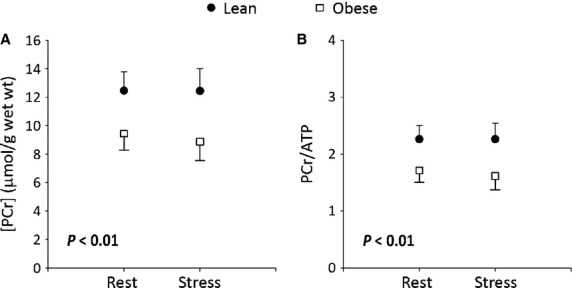
(A) PCr resonance area represented MU. The hearts of diabetic rats had significantly lower [PCr] compared to age‐matched lean rats. (B) PCr/ATP ratio in diabetic myocardium was significantly lower when compared with age‐matched controls. Dobutamine infusion did not result in any changes in [PCr], or PCr/ATP ratio. Unpaired student *t*‐test was used to determine group differences.

Diabetic rats had significantly lower [PCr] (9.43 ± 1.17 vs. 12.46 ± 1.34 *μ*mol/g wet weight; *P* < 0.01) and PCr/ATP ratio (1.71 ± 0.21 vs. 2.26 ± 0.24; *P* < 0.01) than age‐matched lean rats. Dobutamine administration resulted in a ~40% increase in heart rate in both groups but PCr content and the PCr/ATP ratio were not affected (Fig. 3). Myocardial [ATP] was similar between the two groups (*P* = 0.2) whereas [ADP] concentration at baseline were significantly higher in myocardium from obese rats (51.64 ± 5.00 vs. 35.75 ± 5.32 *μ*mol/L; *P* < 0.01).

**Figure 4.**
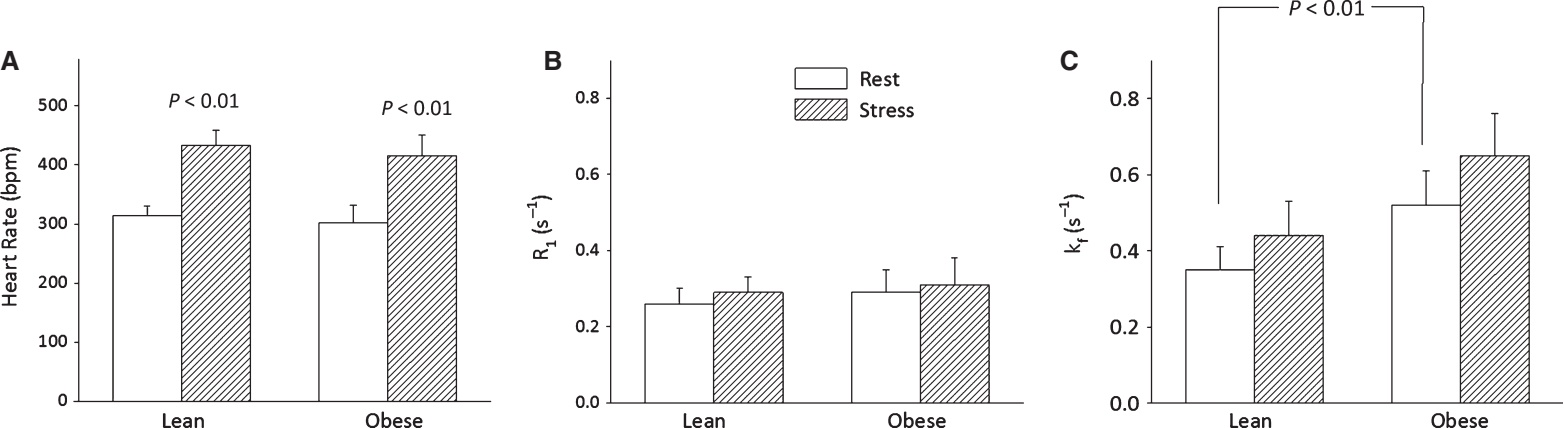
(A) Heart rate at rest was similar between the two groups. Dobutamine caused similar (about 40%) increase in the heart rate in both groups. (B) Intrinsic PCr relaxation rate constant (*R*_1_) was indistinguishable between the groups at rest and stress. (C) Forward CK reaction rate constant (*k*_f_) was significantly higher at rest in diabetic rat heart as determined by unpaired *t*‐test. Number of animals in each group are given in Table 1. Increase in cardiac work resulted in similar increases in *k*_f_ (~0.12 s^−1^) in both groups.

The kinetic data showed significant differences in the forward first‐order rate constant of the CK reaction (*k*_*f*_) between obese and lean rats. At baseline the *k*_*f*_ was significantly higher in diabetic myocardium when compared to the age‐matched lean rats (0.52 ± 0.09 s^−1^ vs. 0.35 ± 0.06 s^−1^; *P* < 0.01). The reaction rate constant increased in both lean (0.35 ± 0.06 s^−1^ to 0.44 ± 0.09 s^−1^; *P* < 0.01) and diabetic (0.52 ± 0.09 s^−1^ to 0.65 ± 0.11 s^−1^; *P* = 0.02) hearts with dobutamine stress. The mean in vivo forward rate of ATP synthesis through cardiac CK at rest was 4.94 ± 1.23 *μ*mol/g/s in obese rats and 4.32 ± 1.05 *μ*mol/g/s in lean rats (*P* = 0.24). *R*_*1*_ for PCr was indistinguishable between the two groups (lean = 0.26 ± 0.04 s^−1^ vs. obese = 0.29 ± 0.06 s^−1^, *P* = 0.22) and did not change with dobutamine stress (Fig. 4).

## Discussion

We demonstrated successful measurement of rate of ATP synthesis through CK in rat model of type II diabetes using noninvasive saturation transfer ^31^P MRS. In this study we found that PCr concentration and PCr/ATP ratio is significantly reduced in obese T2DM rat hearts as compared to those from lean age‐matched control rats. We further found that pseudo‐first‐order forward rate CK reaction rate constant (*k*_*f*_) is higher in ZDF diabetic rat hearts as compared to lean control animals. These results disprove our original hypothesis that CK flux ZDF rat hearts will be reduced. Our results also contradict previous reports of reduced CK reaction kinetics in type I diabetic rat hearts (Popovich et al. 1989; Matsumoto et al. 1995; Spindler et al. 1999). Dobutamine stress resulted in approximately similar increase in *k*_*f*_ in both groups, whereas PCr and ATP levels were maintained.

Oxidative phosphorylation in mitochondria is the primary source of ATP production in the heart. CK reaction is important in energy metabolism as it catalyzes the interconversion of PCr and ADP with Cr and ATP. This facilitates the transfer of high‐energy phosphates from mitochondria (where ATP is produced) to myofibrils (where ATP is consumed) and enables the return of products to mitochondria for rephosphorylation (Lipskaya 2001; Wallimann et al. 2011). In case of impaired oxidative phosphorylation the ATP concentration in the cytosol is maintained at the expense of PCr resulting in a reduced PCr/ATP ratio. The PCr/ATP ratio is therefore routinely used as a marker of myocardial energy balance in research and clinical studies. MRS studies in both type I and type II diabetic human subjects have shown lower PCr/ATP ratio suggesting reduced energy production (Metzler et al. 2002; Scheuermann‐Freestone et al. 2003). In one study the decrease in PCr/ATP was found to be proportional to the degree of diastolic dysfunction, suggesting a role for impaired cardiac mitochondrial energetics (Diamant et al. 2003). To our knowledge, this study is the first to demonstrate reduced [PCr] and PCr/ATP ratio in an animal model of T2DM. These results are in contrast to two studies that reported unchanged PCr/ATP in perfused type I diabetic rat hearts (Matsumoto et al. 1995; Spindler et al. 1999). The difference could be due to the severity, duration, and/or the etiology of diabetes. Our study is in good agreement with several other reports which demonstrated reduced PCr and ATP in STZ (insulin‐deficient type I) diabetic rat hearts (Savabi and Kirsch 1991; Stroedter et al. 1995; Jilkina et al. 2008).

An important finding of this study is that the forward CK reaction rate constant measured in vivo is about 50% higher at baseline in diabetic myocardium when compared to controls. This is in contrast with previous studies which reported about 30% reduction in isolated perfused type I diabetic rat hearts (Matsumoto et al. 1995; Spindler et al. 1999). These differences could be due to several factors. The results obtained in perfused explanted hearts may not reflect the metabolism of the heart in live animals. In particular, the spontaneous heart rate reported in diabetic heart was about 30% lower and the rate pressure product (RPP) was about half that found in control hearts (Jilkina et al. 2008; Miki et al. 2013). This could account for the reduced CK flux since CK reaction kinetics are coupled to workload (Bittl and Ingwall 1985). In addition, in these previous studies the inorganic phosphate concentration ([Pi]) was more than double in the diabetic myocardium relative to healthy hearts. High [Pi] has been associated with ischemia and a fall in developed pressure, which could reduce ATP production via CK (Elliott et al. 1994; He et al. 1997). Finally, the free energy of ATP hydrolysis is proportional to log[(ATP)/(ADP)x(Pi)]; therefore the high levels of Pi may reflect a low‐energy state in these perfused hearts.

Several studies using biochemical assays have reported decreased CK activity and altered isoenzyme distribution in streptozotocin (STZ)‐treated rat hearts (Khuchua et al. 1989; Popovich et al. 1989; Awaji et al. 1990; Spindler et al. 1999). Reduced CK activity would mean lower *k*_*f*_, whereas we observed increased *k*_*f*_ in this study. However, in a recent study Lin et al. (2009) examined the regulation of M‐CK isoenzyme in diabetic rat hearts. They reported that although the content of CK isoenzyme was reduced by 34% the phosphorylation of M‐CK was reduced by 71% in diabetic hearts compared to controls, much greater than the decrease in the expression of enzyme. As a result, the forward activity of CK reaction was increased by about 70% in hearts from diabetic rats. A 50% increase in CK activity was also reported in left ventricles of 8‐week diabetic rat hearts (Somjen et al. 2006). These reports are in agreement with our results where *k*_*f*_ is increased by about 50% at baseline in diabetic heart. One possible explanation for the reduced total CK activity observed in some previous studies (Khuchua et al. 1989; Popovich et al. 1989; Awaji et al. 1990; Spindler et al. 1999) might be due to the use of different biochemical assays in which reverse CK activity could have been measured.

Interestingly, whereas the CK reaction rate constant was different the diabetic and control hearts, CK flux was similar in the two groups due to a reduced [PCr] concentration in the diabetic hearts. Reduced [PCr] might indicate mitochondrial dysfunction, as previous studies in diabetic rodent hearts have demonstrated reduced mitochondrial respiration and ATP synthesis (Kuo et al. 1983, 1985; Boudina et al. 2005). The heart rate in the two groups was indistinguishable at baseline indicating comparable baseline workload therefore the increase in *k*_*f*_ might represent a mechanism to maintain cytosolic ATP at the expense of PCr. Both groups responded likewise to the high workload with similar increase in the heart rate and CK reaction rate constant. The [PCr] was also indistinguishable pre‐ and postdobutamine infusion in control heart. These results indicate that under these conditions CK system does not limit the ability to generate ATP for cardiac work.

In line with diabetic rat heart myocardium contradictory results have been reported in CK activity in pressure in left ventricle after pressure overload. A recent study using ^31^P MR spectroscopy demonstrated ~30% reduction in *k*_*f*_ and ~50% reduction in CK flux in heart failure caused by pressure overload after 7 to 8 weeks of thoracic aortic constriction (Gupta et al. 2011). In contrast, two previous studies showed increase in steady‐state levels of M and B creatine kinase subunit and also mitochondrial creatine kinase mRNA and concomitant increase in total CK activity (Meerson and Javich 1982; Fontanet et al. 1991). These changes were observed early after aortic banding, before left ventricle hypertrophy ensued. Studies with longer term exposure to pressure showed normal CK activity (Vatner and Ingwall 1984; Younes et al. 1985; Pauletto et al. 1989). Although the reasons for these difference have not been examined but it can be speculated that initial increased energy requirements in pressure overload leads to increased CK activity as an adaptive response to hemodynamic stress. As hypertrophy ensues the CK enzyme activity decreases toward control levels. When heart failure develops the CK flux regresses further and may lead to deficit in ATP delivery especially at high workloads. A similar time course in CK activity can be speculated in case of DCM. Numerous metabolic and pathophysiological stimuli are involved in its development finally leading to systolic and diastolic left ventricular (LV) dysfunction (Fang et al. 2004; Voulgari et al. 2010). An early increase in CK flux could be a compensatory mechanism to support initial metabolic and structural remodeling of myocardium eventually becoming maladaptive and leading to heart dysfunction.

There are several limitations that need mentioned. We did not measure heart function in this study therefore cannot draw any conclusions about the relationship between the CK reaction kinetics and cardiac function. Previous studies have reported enhanced, similar, and reduction in heart function in ZDF rats (Fredersdorf et al. 2004; van den Brom et al. 2010; Daniels et al. 2012). The reasons for these differences are not clear perhaps duration of diabetes, experimental conditions, and anesthetic mechanism could all potentially contribute to these differences. We also did not measure blood pressure in these rats and therefore could not calculate rate pressure product as an indicator of cardiac work. However, the heart rate of the two groups responded similarly to dobutamine administration, making it reasonable to assume that the workload was elevated similarly. Additionally, no direct biochemical estimates of CK isoenzyme concentrations were made. Surprisingly reports of CK isoenzyme concentration/activity in ZDF rats hearts are lacking therefore we cannot comment on the relationship between the CK isoenzymes and CK flux measured by ^31^P MRS. Additional time course studies determining the direct relationship between CK isoenzymes, CK flux, and heart function would be needed to answer these questions.

From an experimental perspective it is conceivable that there could be contaminating signal arising from other tissues (i.e., stomach and gut). To get assurance that this contamination was small we did imaging experiments with a similar sized proton coil and the signal was primarily restricted to the heart. We cannot rule out presence of lung in the sensitive area of RF coil that does not contribute to ^31^P signal. As a consequence we were not able to directly quantify PCr and ATP concentrations in the heart. However, PCr/ATP ratio reported uses ATP as an internal standard and thus is independent of different contribution volumes and coil loading. In ^31^P MRS the most commonly used reference is in vivo [ATP], which has been shown to be very consistent across species and ATP levels remain normal in myocardium until advanced stages of heart failure (Neubauer 1999; Hitchins et al. 2001; Kemp et al. 2007; El‐Sharkawy et al. 2013). We have also employed the use of this standard assumption to calculate in vivo metabolite concentrations and this assumption is justified given the wealth of published reports.

In summary, the present study demonstrates that the PCr content is reduced in type II diabetic rat hearts, indicative of impairment in mitochondrial ATP production. The forward CK reaction rate constant is elevated, possibly reflecting a compensatory mechanism to support increased flux through the CK shuttle needed to support cardiac work. We did not observe any fall in PCr content with increased cardiac work. CK reaction velocity increased in both diabetic and control hearts to maintain constant ATP content at higher work.

## Acknowledgments

The authors would also like to thank John Engelbach for help with animal experiments.

## Conflict of Interest

None declared.
